# Divergent trends in lung cancer incidence by gender, age and histological type in Estonia: a nationwide population-based study

**DOI:** 10.1186/s12885-017-3605-x

**Published:** 2017-08-30

**Authors:** Tiiu Aareleid, Mari-Liis Zimmermann, Aleksei Baburin, Kaire Innos

**Affiliations:** 1 Department of Epidemiology and Biostatistics, National Institute for Health Development, Hiiu 42, 11619 Tallinn, Estonia; 2 Estonian Cancer Registry, National Institute for Health Development, Hiiu 42, 11619 Tallinn, Estonia

**Keywords:** Lung cancer, Population-based, Incidence, Mortality, Gender, Age, Histology, Time trends, Predictions

## Abstract

**Background:**

Lung cancer (LC) is the leading cause of cancer deaths in men and the second most frequent cause of cancer deaths in women in Estonia. The study aimed to analyze time trends in LC incidence and mortality in Estonia over the 30-year period, which included major social, economic and health care transition. The results are discussed in the context of changes in tobacco control and smoking prevalence. Long-term predictions of incidence and mortality are provided.

**Methods:**

Data for calculating the incidence and mortality rates in 1985–2014 were obtained from the nationwide population-based Estonian Cancer Registry and the Causes of Death Registry. Joinpoint regression was used to analyze trends and estimate annual percentage change (APC) with 95% confidence interval (CI). Nordpred model was used to project future incidence and mortality trends for 2015–2034.

**Results:**

Incidence peaked among men in 1991 and decreased thereafter (APC: -1.5, 95% CI: -1.8; −1.3). A decline was seen for all age groups, except age ≥ 75 years, and for all histological types, except adenocarcinoma and large cell carcinoma. Incidence among women increased overall (APC: 1.6, 95% CI: 1.1; 2.0) and in all age groups and histological types, except small cell carcinoma. Age-standardized incidence rate (world) per 100,000 was 54.2 in men and 12.9 in women in 2014. Changes in mortality closely followed those in incidence. According to our predictions, the age-standardized incidence and mortality rates will continue to decrease in men and reach a plateau in women.

**Conclusions:**

The study revealed divergent LC trends by gender, age and histological type, which were generally consistent with main international findings. Growing public awareness and stricter tobacco control have stimulated overall favorable changes in men, but not yet in women. Large increase in incidence was observed for adenocarcinoma, which in men showed a trend opposite to the overall decline. LC will remain a serious public health issue in Estonia due to a high number of cases during the next decades, related to aging population, and previous and current smoking patterns. National tobacco control policy in Estonia should prioritize preventing smoking initiation and promoting smoking cessation, particularly among women.

## Background

Lung cancer (LC) is the leading cause of cancer deaths in men and the second most frequent cause of cancer deaths in women in Estonia [1]. There is convincing epidemiological evidence that cigarette smoking is the main risk factor of LC and thus, tobacco control plays the primary role in LC prevention. Other confirmed risk factors of LC include indoor and outdoor air pollution (including secondhand tobacco smoke), exposure to hazardous chemicals, radiation, asbestos, silica dust and several elements, including arsenic [2].

In 2012, high LC incidence in men was estimated for the countries of Central and Eastern Europe, but the reverse situation was described among women: incidence was low in Eastern Europe and high in Northern and Western Europe [3]. Geographical patterns of mortality closely correspond to those of incidence. In Europe overall, declining trend among men and increasing trend among women has been observed [4]. Time trends in incidence vary by histological type [5]. A worldwide increase for adenocarcinoma and decrease for squamous cell carcinoma and small cell carcinoma has been reported [6].

In a previous study, we analyzed LC occurrence in Estonia in the Soviet era when neither tobacco-control legislation nor organized public health measures for reducing risk of tobacco-related diseases were in place [7]. This study aims to analyze trends in LC by gender, age and main histological types during and after social, economic and health care transition, and discuss the findings in the context of changes in tobacco control and smoking prevalence. We also provide predictions of incidence and mortality up to 2034.

## Methods

Data on incident cases of LC (ICD-10 C33 − C34) were obtained from the Estonian Cancer Registry that is a population-based registry with nationwide coverage since 1968. Reporting of cancer cases to the registry is mandatory by law for all physicians and pathologists in Estonia. The registry uses multiple sources for case ascertainment, including trace-back of cases first identified via death certificates and linkages with the electronic patient records of two cancer centres. The quality of the cancer registry data has been relatively high [8] and comparable with that shown for other registries participating in international projects [9]. The population of Estonia was 1.34 million according to the 2011 census. LC mortality data were obtained from the Estonian Causes of Death Registry and population denominators from Statistics Estonia. Incidence and mortality rates were age-standardized to world standard population [9].

Age-specific incidence and mortality rates were calculated for the age-groups 15–54, 55–64, 65–74 and ≥75 years. Age-standardized incidence rates were calculated for four major histological groups as defined in the Cancer in Five Continents, vol. X [9]: (1) squamous cell carcinoma (SQC; ICD-O-3 morphology codes 8050–8078, 8083–8084; (2) adenocarcinoma (ADC; 8140, 8211, 8230–8231, 8250–8260, 8323, 8480–8490, 8550–8551, 8570–8574, 8576); (3) small cell carcinoma (SMC; 8041–8045); (4) large cell carcinoma including giant cell, clear cell and large cell undifferentiated carcinoma (LCC; 8010–8012, 8014–8031, 8035, 8310).

Joinpoint analysis with Joinpoint Regression Program (version 4.1.1.1) from the Surveillance Research Program of the US National Cancer Institute (http://surveillance.cancer.gov/joinpoint/) was performed to model the rates and calculate the estimated annual percentage change (APC) for incidence and mortality trends. Joinpoint regression identifies points of significant change in the log linear slope of the trend. The optimal number of joinpoints was selected with permutation test. For each line segment separated by joinpoints, the APC was presented with 95% confidence intervals (CI). All analyses were done separately for men and women. We modelled age-standardized rates for total LC and four histological groups, and crude rates for age-specific analyses.

The five-year LC incidence and mortality rates from 2015 to 2019 to 2030–2034 were projected using the Nordpred model [10]. The prediction was based on cases observed during 1985–2014. Population denominator data for 1985–2014 and projected numbers for 2015–2034 were obtained from Statistics Estonia [11]. Data were grouped into five-year calendar periods and five-year age groups. To forecast LC incidence and mortality for 2015–2034, R-based Nordpred software was used with power link function age-period-cohort model [10]. The lower age limit was based on a minimum of 10 cases in all observation periods (30 years for incidence and 35 years for mortality).

The study protocol was approved by the Tallinn Medical Research Ethics Committee (decision no. 284). We used registry data with no personal identifiers. These data were either public or available for research upon request without permission.

## Results

### Patients

A total of 22,890 new LC cases (18,399 in men and 4491 in women) were diagnosed and 20,219 deaths (16,420 in men and 3799 in women) were registered in Estonia in 1985–2014. The proportion of microscopically verified cases significantly increased over time and reached 77% for men and 75% for women in 2010–2014 (Table 1). For both genders, the proportion of death certificate only cases (%DCO) increased in the early 2000s and remained around 4% thereafter. Proportion of cases diagnosed at autopsy decreased and was 2% in 2010–2014*.* Percentage of patients aged ≥75 years increased 2.3-fold among men and 1.7-fold among women from 1985–1989 to 2010–2014.

**Table 1:** Incident cases of lung cancer, Estonia 1985–2014

	Period of diagnosis						*p*-value^a^
	1985–89	1990–94	1995–99	2000–04	2005–09	2010–14	
*Men*							
Total number of cases	2912	3232	3200	3054	2975	3026	
Microscopic verification (%)	70.8	72.5	73.6	74.1	72.3	76.6	*p* < 0.001
Death certificate only (%)	0.2	1.2	1.6	4.5	4.3	3.8	*p* < 0.001
Autopsy cases (%)	7.2	5.2	3.3	3.3	3.0	2.1	*p* < 0.001
Morphology (%)^b^							
Small cell carcinoma	24.2	23.7	20.8	21.3	19.6	16.4	*p* < 0.001
Squamous cell carcinoma	39.0	45.3	49.7	49.1	46.4	42.0	*p* = 0.04
Adenocarcinoma	9.7	8.0	10.0	12.1	15.7	23.0	*p* < 0.001
Large cell carcinoma	6.2	6.3	7.3	9.5	11.0	11.1	*p* < 0.001
Other specified	0.9	0.9	1.1	1.9	2.6	4.8	*p* < 0.001
NOS	20.1	15.9	11.2	6.2	4.7	2.7	*p* < 0.001
Age at diagnosis (%)							
15–54 years	19.8	18.1	14.3	12.2	10.4	7.6	*p* < 0.001
55–64 years	43.3	40.9	35.3	27.7	24.9	27.3	*p* < 0.001
65–74 years	24.8	30.2	39.7	42.2	39.8	36.8	*p* < 0.001
≥75 years	12.1	10.8	10.8	17.9	24.9	28.3	*p* < 0.001
Median age at diagnosis (years)	61	63	65	67	68	69	
*Women*							
Total number of cases	582	601	678	766	805	1059	
Microscopic verification (%)	59.0	60.6	63.0	68.4	68.1	74.6	*p* < 0.001
Death certificate only (%)	0.2	1.7	2.2	4.3	4.6	3.5	*p* < 0.001
Autopsy cases (%)	10.1	7.8	3.4	3.9	2.0	2.1	*p* < 0.001
Morphology (%)^b^							
Small cell carcinoma	26.2	27.5	24.8	21.6	19.3	13.5	*p* < 0.001
Squamous cell carcinoma	22.5	21.7	25.3	28.1	22.8	22.0	*p* = 0.87
Adenocarcinoma	19.8	20.3	22.5	28.4	34.5	40.3	*p* < 0.001
Large cell carcinoma	8.5	8.5	8.9	12.0	11.9	12.4	*p* = 0.05
Other specified	1.8	1.7	4.7	4.0	5.1	9.9	*p* < 0.001
NOS	21.3	20.3	13.8	5.9	6.4	1.9	*p* < 0.001
Age at diagnosis (%)							
15–54 years	12.9	11.3	11.4	12.3	9.8	8.4	*p* < 0.001
55–64 years	32.5	25.0	17.7	21.0	22.5	21.6	*p* < 0.001
65–74 years	33.0	39.9	43.8	32.6	30.1	33.2	*p* = 0.92
≥75 years	21.7	23.8	27.0	34.1	37.6	36.7	*p* < 0.001
Median age at diagnosis (years)	65	68	69	71	70	71	

^a^Comparing proportions for the first and last period

^b^Among microscopically verified cases

The most common histological types overall were SQC among men and ADC among women. In both genders, the proportion of ADC cases substantially increased over time. The proportion of cases with unspecified morphology dropped about 10-fold from the first to the last period.

### Time trends

**Fig. 1:**
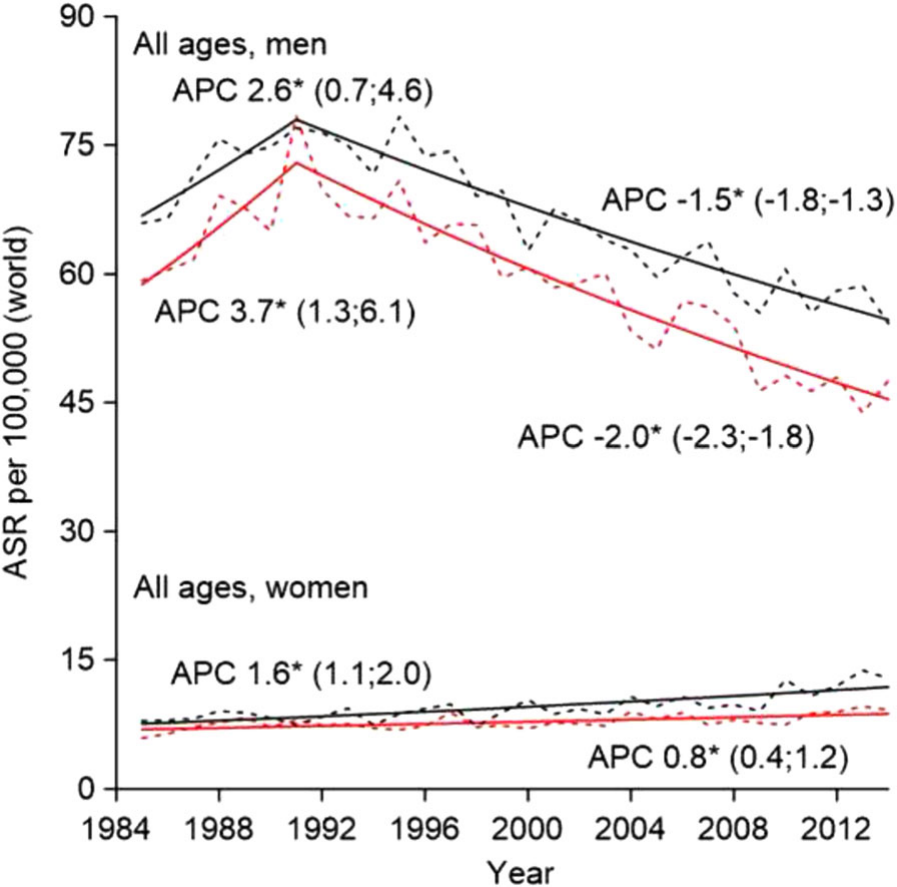
Observed (dotted line) and modeled (solid line) age-standardized rates (ASR) and annual percentage change (APC) for trends in incidence (black line) and mortality (red line) of lung cancer in Estonia, 1985–2014. *The APC is significantly different from zero at alpha = 0.05

Age-standardized incidence rate (ASIR) peaked among men in 1991 and turned to decline thereafter (APC: -1.5, 95% CI: -1.8; −1.3) (Fig. 1). Among women, incidence steadily increased over the entire study period (APC: 1.6, 95% CI: 1.1; 2.0). ASIR per 100,000 was 54.2 in men and 12.9 in women in 2014. The difference between annual incidence and mortality rates slightly increased over the study period. The age standardized mortality rate (ASMR) per 100,000 was 47.6 in men and 9.2 in women in 2014.

**Fig. 2:**
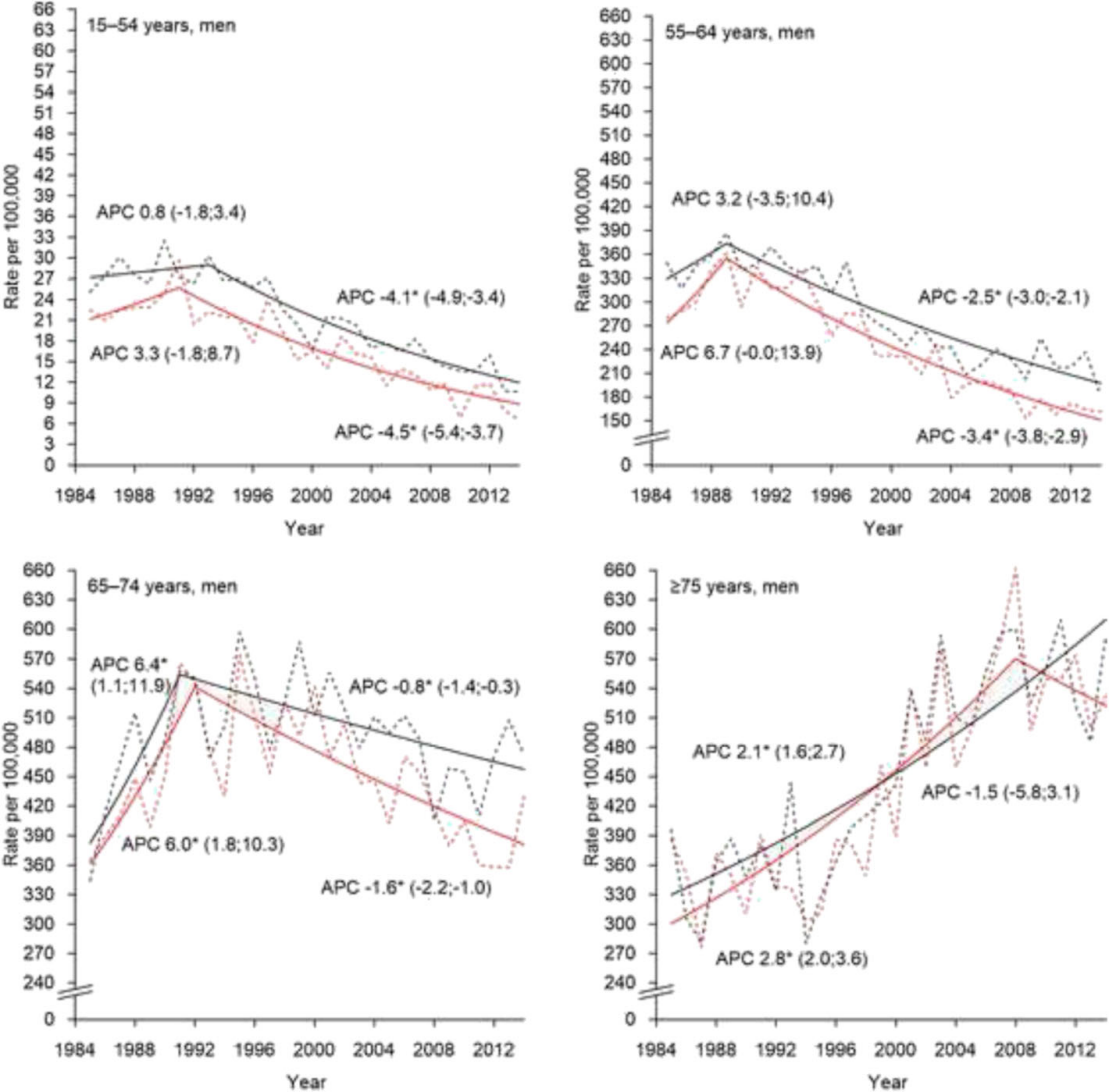
Observed (dotted line) and modeled (solid line) age-specific rates and annual percentage change (APC) for trends in incidence (black line) and mortality (red line) of lung cancer in Estonia, men 1985–2014. *The APC is significantly different from zero at alpha = 0.05

**Fig. 3:**
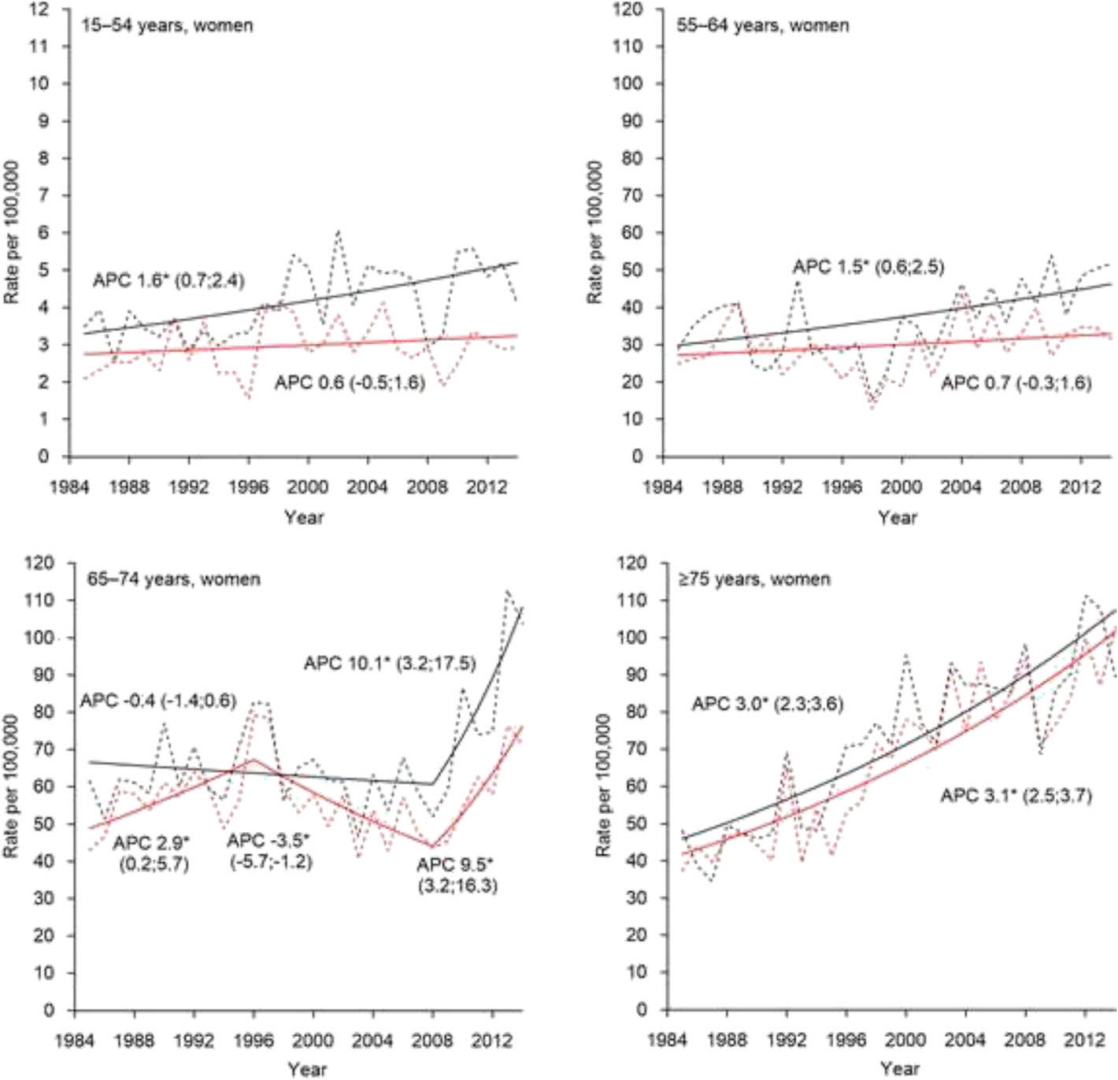
Observed (dotted line) and modeled (solid line) age-specific rates and annual percentage change (APC) for trends in incidence (black line) and mortality (red line) of lung cancer in Estonia, women 1985–2014. *The APC is significantly different from zero at alpha = 0.05

Among men, both incidence and mortality have been in steep decline since the early 1990s for all age groups except ≥75 years, where a significant rise continued over the entire study period (Fig. 2). Among women aged 15–54 and 55–64 years, significant but relatively modest increases in incidence were seen, while the corresponding changes in mortality were not significant (Fig. 3). In women aged 65–74 years, incidence and mortality turned to a rapid increase from the end of the 2000s. The steepest long-term rise was observed among women aged ≥75 years.

**Fig. 4:**
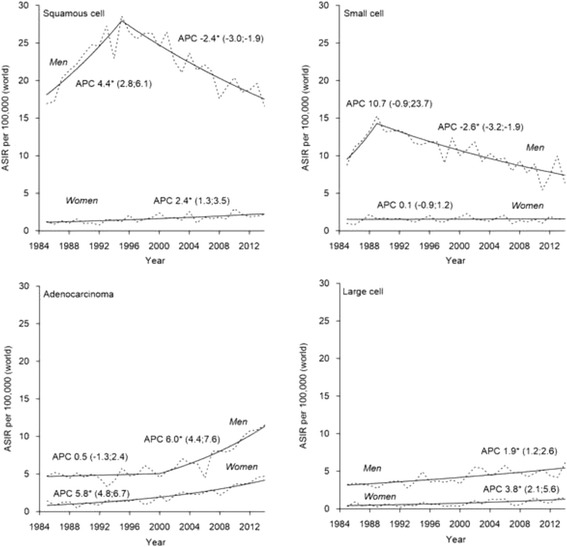
Observed (dotted line) and modeled (solid line) age-standardized incidence rates (ASIR) and annual percentage change (APC) for trends of lung cancer by histological type in Estonia, 1985–2014. *The APC is significantly different from zero at alpha = 0.05

In men, the incidence trends of SQC and SMC generally followed the overall trend, while an increase was observed for ADC after 2000 and for LCC over the whole study period (Fig. 4). In women, incidence increased for SQC, ADC and LCC, but remained stable for SMC.

### Future predictions

**Fig. 5:**
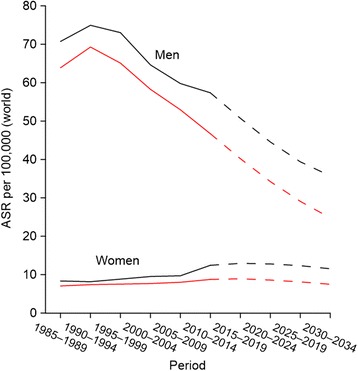
Observed (solid line) and predicted (dashed line) age-standardized incidence (black line) and mortality (red line) rates (ASR) of lung cancer in Estonia

According to our prediction, the age-standardized incidence and mortality will continue to decrease among men (predicted ASIR and ASMR for 2030–2034 are 36 cases and 25 deaths per 100,000, respectively) (Fig. 5). Respective rates will stabilize in women (12 cases and 7 deaths per 100,000). The predicted average annual number of new cases for 2030–2034 is 492 in men (113 cases less than in 2010–2014) and 249 in women (37 cases more than in 2010–2014).

## Discussion

This was the first study analyzing LC trends during and after major political, social and economic changes in Estonia. Divergent trends were observed in LC incidence by gender, histology and age. In men, incidence decreased overall and in all age groups except the oldest (≥75 years), and for all histological types except ADC and LCC. In women, however, rise in incidence was seen overall as well as in all subgroup analyses, except for SMC.

The main strength of the study was the availability of uniformly collected nationwide incidence data for 30 years. This was the first study predicting LC incidence and mortality in Estonia, but as a limitation, the modeled predictions were based on historical trends and not on the changes in risk patterns. No individual data were available on smoking history. Another limitation of the study was the high proportion of cases with unspecified morphology during the early periods, which may have affected histology-specific trends. The proportion of microscopically verified cases remained slightly below that of many other European countries [9] and thus, the incidence may include some cases of metastatic disease erroneously attributed to lung as a primary site. The increase in the %DCO was partly due to the growing age of incident cases - this proportion was shown to be considerably higher among cases diagnosed at older age, particularly in age group ≥75 years [12, 13]. The sudden rise in %DCO around 2000 was likely related to a temporary disruption of the case ascertainment practices of the cancer registry - the 2003 Data Protection Act prohibited the use of death certificates as an additional source for case ascertainment [8]. Although the legal problems were solved with the 2007 Act, the trace-back done several years later was not as successful as previously [8].

Trends in LC incidence and mortality rates in Estonia were similar to those reported in other developed countries: peaking followed by a sharp decrease in men, a steady increase in women and a narrowing gap between the genders [14]. As survival from LC is generally poor, the changes in mortality closely followed those in incidence, but increasing difference between incidence and mortality rates reflects improvements in survival observed in Estonia, particularly among younger patients [12]. In 2012, the ASIR in Estonia was slightly above the estimated all-European level in men, but notably below that in women [15].

Upward trends in female LC rates have been observed in most European countries. In the EU as whole, the ASMR (world) increased 2.3% per year in 2000–2009 and further increase was predicted to reach 14 deaths per 100,000 women in 2015 [4]. Stabilization has occurred in the UK, Iceland and Ireland where mortality peaked already in the mid/late 1990s [16]. Among younger women (age 20–44 years), mortality leveled off or turned to decline in most European countries, suggesting more favorable overall trends in the future [16]. According to our prediction, the ASMR (world) in women in Estonia is currently peaking and expected to decline to about 7 per 100,000 in the 2030s. Thus, despite of a steady rise over several decades, female LC rates in Estonia will probably not reach the high rates reported in some Nordic and Western European countries, nor those predicted for the EU.

Tobacco is the principal established risk factor of LC and trends in LC largely correspond to changes in smoking prevalence [2]. During the Soviet period, people in Estonia consumed mainly domestic cigarettes and *papirossi* (non-filter Russian cigarettes), but also the brands imported from other Eastern European countries. The price of tobacco products was low, but the tar and nicotine levels were high, compared with western products [17]. From 1968 to 1972 to 1983–1987, the ASIR (world) increased 22% among men and 34% in women and the incidence clearly shifted towards younger birth cohorts, with the most expressed rise in age group 45–64 years [7].

After transition to open market economy in the early 1990s, international tobacco brands became available and replaced domestic products in Estonia. National tobacco control policy started with the first Tobacco Act (2000), which included tobacco advertising and price regulations, and smoking restrictions for workplaces and public premises. Tobacco advertising and promotion were substantially restricted by the Advertising Act (2008). Estonia joined the EU in 2004, but the increase of tobacco excise tax started to meet the EU requirements already prior to accession. Taxation in combination with other tobacco control measures led to about 10% decline of total annual cigarette consumption in Estonia in 2004–2011 [18]. In 2016, new changes in the tobacco legislation regulated the appearance of cigarette packages, aiming to reduce the appeal of tobacco products. An important shift in public health attitudes and behaviors has taken place in Estonia in the past few decades. In 2014, about 80% of adult respondents agreed that national comprehensive tobacco control policies are important in order to reduce smoking [19].

**Fig. 6:**
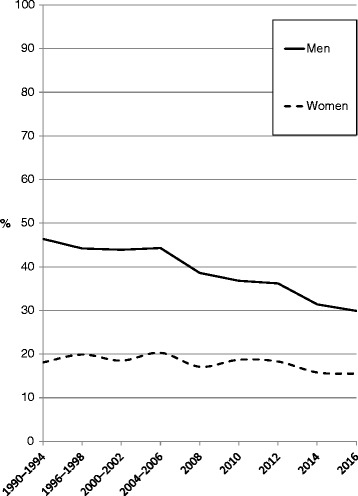
Prevalence of daily smoking (%) among adult population (age 16–64 years) in Estonia, 1990–2016 [22]

Only very limited data are available on smoking prevalence in Estonia during the Soviet time. In the 1980s, an estimated 50% of men and 20% of women were daily smokers [19]. It is likely that smoking prevalence in men peaked in the 1960s or 1970s at an even higher level. For example in Finland, data are available from 1960 when 58% of men were daily smokers [20]. Systematic collection of health behavior information started in Estonia in the 1990s. It has been shown that daily smoking patterns among the Estonian adult population generally fit the model of tobacco epidemic in developed countries [21]. The prevalence of daily smoking among men aged 16–64 years has dropped from 46% in early 1990s to 30% in 2016, while only a slight change has occurred among women (from 18% to 16%) [22] (Fig. 6). Most pronounced decline in daily smoking prevalence has occurred among men aged 16–34 years and among women aged 25–44 years. In Finland, daily smoking prevalence in men started to decline already before the tobacco-control legislation came into force in 1976, and reached 17% in 2014 [20]. Smoking prevalence in women since the 1990s has been similar in Finland (14% in 2014) and Estonia. It is noteworthy that male LC incidence rates in Estonia and Finland were close in the mid-1980s, after which a rapid decline started in Finland and despite the decline in Estonia since the 1990s, male incidence currently remains almost twice the rate in Finland [23]. On the other hand, female LC rates and time trends have been very similar in both countries throughout the 30-year period.

Large educational inequalities, particularly among women, have been found in smoking-related mortality in Europe [24] and education is an important determinant of daily smoking [20, 25, 26]. In 2016, the proportion of daily smokers in Estonia ranged from 15% among men with higher education to 44% among those with basic education, while among women, the respective proportions were 6% and 29% [22]. During 1990–2010, the prevalence of daily smoking doubled among women with basic education [21], which may have contributed to the observed increase in incidence.

Age at initiation of smoking is another important determinant of LC trends [27]. According to the results of an international health behavior study in 2013/2014, Estonia ranked third among 45 countries after Greenland and Lithuania with regard to the percentage of schoolchildren reporting first smoking experience at age 13 or younger [28]. Daily smoking prevalence among 15–16-year-old boys has decreased twofold in Estonia since 1995, reaching 13% in 2015, whereas the change was much smaller among girls (from 13% to 10%) [29]. This indicator is probably a major determinant of future LC trends beyond our prediction period. As shown in a recent global overview on smoking prevalence, preventing children, adolescents and young adults from starting to smoke is one of the main tasks of tobacco control today [30].

In 2016, the vast majority of adult daily smokers (95% men and 97% women) in Estonia used filter cigarettes and the use of non-filter cigarettes has declined dramatically since the 1990s [22]. At the same time, smokeless tobacco products like snus and e-cigarettes have become more popular, although snus is prohibited in Estonia. Among men aged 16–24 years, the daily use of e-cigarettes increased from 0.4% in 2012 to 5% in 2016 [22, 31]. Daily use of e-cigarettes and snus was reported by 3% of 15-year-old boys in 2013/2014 [28]. E-cigarettes have been shown to be less toxic than conventional cigarettes, but the amount of risk reduction is yet unknown [32]. This might be a topic to consider because the use of snus and e-cigarettes in younger age groups might influence future LC trends.

Time trends by main histological types in Estonia followed international patterns. ADC incidence is still increasing among women in most countries, but has begun to stabilize among men [5, 6]. The reasons for worldwide increase in ADC incidence remain unclear. Historically, ADC has been the most common cell type of LC in women and in non-smoking men [33]. It has been suggested that the increasing use of filtered low tar cigarettes is associated with an increased risk of ADC and elevated ADC/SQC ratio [34]. However, several studies do not support this hypothesis: there is a clear increase in the ADC/SQC ratio in never smokers that cannot logically arise from changes in cigarette design [35]. The global findings from gene expression analyses may provide improved understanding of biological pathways of the development of lung ADC, regardless of patients’ smoking status [36]. It has also been suggested that refinements of histological techniques and differential use of nonspecific morphology codes may have caused artificial fluctuations in the incidence rates for histological subtypes, thus biasing temporal trends [37] and leading to an increase in ADC diagnosis [33]. In this study we observed about 10-fold decrease in the proportion of cases with non-specified morphology over the 30-year period in Estonia, which reflects major improvements in histopathological diagnosing.

From 1990 to 2014, life expectancy at birth in Estonia increased from 65 to 72 years for men and from 75 to 82 years for women [11]. Due to the increasing proportion of elderly people, crude incidence and mortality trends will not be as favorable as age-standardized trends. In women, the absolute number of LC cases and deaths will continue to increase. The effect of aging population is well illustrated by the substantial increase of median age at diagnosis and trends observed in the oldest age groups.

## Conclusion

The study revealed divergent trends in LC incidence and mortality by gender, age and histological type in Estonia, which were generally consistent with main international findings. Diverging gender-specific trends are probably related to different trends in smoking. Growing public awareness and stricter tobacco-control strategies have stimulated overall favorable changes in smoking prevalence and LC occurrence in men. In women, however, a significant increase in LC incidence was seen in all age groups. Among histological types, the most pronounced increase in incidence was observed for ADC, which in men showed a trend opposite to the overall declining incidence. This warrants further monitoring of LC trends by histology. According to our predictions, the age-standardized incidence and mortality rates will continue to decrease in men and reach a plateau in women during next decades. Nevertheless, LC will remain a serious public health issue due to a high number of cases at least during the next decades, related to aging population and to previous and current smoking patterns. National tobacco control policy in Estonia should prioritize preventing smoking initiation among children and young adults, and promoting smoking cessation in all age groups. Special efforts in should be addressed to women and population groups with lower education.

## Abbreviations

%DCO: Proportion of Death Certificate Only Cases
ADC: Adenocarcinoma
APC: Annual Percentage Change
ASIR: Age-Standardized Incidence Rate
ASMR: Age-Standardized Mortality Rate
ASR: Age-Standardized Rate
CI: Confidence Interval
LC: Lung Cancer
LCC: Large Cell Carcinoma
NOS: Not Otherwise Specified
SMC: Small Cell Carcinoma
SQC: Squamous Cell Carcinoma
